# Conduct Problem Trajectories Between Age 4 and 17 and Their Association with Behavioral Adjustment in Emerging Adulthood

**DOI:** 10.1007/s10964-016-0476-4

**Published:** 2016-03-26

**Authors:** Miranda Sentse, Tina Kretschmer, Amaranta de Haan, Peter Prinzie

**Affiliations:** 1 Department of Psychology, Education and Child Studies, Erasmus University Rotterdam, P.O Box 1738, 3000 DR Rotterdam, The Netherlands; 2 Department of Psychiatry, University Medical Center Groningen, Groningen, The Netherlands; 3 Department of Pedagogy and Educational Sciences, University of Groningen, Groningen, The Netherlands

**Keywords:** Conduct problems, Trajectories, Antisocial behavior, Mixture modeling, Emerging adulthood

## Abstract

Individual heterogeneity exists in the onset and development of conduct problems, but theoretical claims about predictors and prognosis are often not consistent with the empirical findings. This study examined shape and outcomes of conduct problem trajectories in a Belgian population-based sample (*N* = 682; 49.5 % boys). Mothers reported on children’s conduct problems across six waves (age 4–17) and emerging adults reported on their behavioral adjustment (age 17–20). Applying mixture modeling, we found four gender-invariant trajectories (labeled life-course-persistent, adolescence-onset, childhood-limited, and low). The life-course-persistent group was least favorably adjusted, but the adolescence-onset group was similarly maladjusted in externalizing problems and may be less normative (15 % of the sample) than previously believed. The childhood-limited group was at heightened risk for specifically internalizing problems, being more worrisome than its label suggests. Interventions should not only be aimed at early detection of conduct problems, but also at adolescents to avoid future maladjustment.

## Introduction

Onset and development of conduct problems are characterized by substantial individual heterogeneity. Moffitt (1993) proposed a taxonomy of conduct problems according to age of onset by distinguishing life-course-persistent from adolescence-limited conduct problems and hypothesized different etiologies and outlooks to master transition into adulthood for these two types. That is, life-course-persistent conduct problems emerge early in life and persist over time as a result of negative person–environment transactions (Moffitt 1993). The theory proposes that child risk factors such as neuropsychological problems, hyperactivity, and a difficult temperament are inherited or developed early in life, and further exacerbated by environmental risk factors like negative parenting, low socioeconomic status, and parental divorce. Cumulating personal- and environmental risks over time are hypothesized to create difficulties in multiple aspects of adult life. Adolescence-limited conduct problems, on the other hand, emerge in adolescence and result from discordance between biological maturation and access to adult privileges, also known as the “maturity gap”. Here conduct problems can be seen as a means to challenge the rules by authority figures and to gain a sense of autonomy. Therefore, at this age conduct problems are considered normative and, in contrast to the life-course-persistent path, limited to the adolescent years (Moffitt 1993).

The distinction between life-course-persistent and adolescence-limited conduct problems has been supported by multiple studies (e.g., Broidy et al. 2003; Nagin and Land 1993). Nonetheless, in an extensive review of these studies, Moffitt et al. (2008) pointed at several unanswered issues. First, multiple studies found some youth with a child-onset of conduct problems who did not continue with their conduct problems into late adolescence, which suggests a childhood-limited conduct problems pathway (Barker and Maughan 2009; Odgers et al. 2007, 2008). Moreover, Moffitt et al. (2008) concluded that there is no consensus about adult adjustment of these children. Second, adolescence-onset antisocial behavior is not necessarily, or even rarely, limited to adolescence and can act as a marker for future maladjustment in the externalizing spectrum (Fairchild et al. 2013; Kretschmer et al. 2014; Nagin et al. 1995). In other words, the adolescence-limited label may be misleading and should instead be referred to as adolescence-onset. One can also speculate about the normativity of this trajectory, given that in many studies this group is not that large (see e.g., Van Dulmen et al. 2009).

Intrigued by these controversies between theoretical claims and empirical findings, the current study aims to answer two questions related to the conduct problem trajectories. First, are conduct problems specific to a developmental period as claimed for adolescence-limited by Moffitt and for childhood-limited suggested by others (e.g., Barker and Maughan 2009; Odgers et al. 2007, 2008)? Resolving this question is not easy as the existing studies (see for reviews Moffitt et al. 2008; Piquero 2008; Van Dulmen et al. 2009) differ considerably in design and analytical strategy. Most importantly, the age-span that is covered by the trajectories differs among the studies, and many studies did not include children young enough to detect a childhood-limited trajectory (e.g., Piquero et al. 2005) or children old enough to draw conclusions about the adolescence-limited trajectory (e.g., Barker and Maughan 2009). In addition, many studies are limited to male-only and/or high-risk non-European samples (e.g., Moffitt et al. 1996; Odgers et al. 2007; Roisman et al. 2004) questioning the applicability of these trajectories to population-based European samples and to girls. Last, some studies have used conventional statistical methods that fail to respond to individual heterogeneity in developmental patterns by using arbitrary, manually constructed cut-offs to create the groups (e.g., Moffitt et al. 1996; Roisman et al. 2004). Thus, to answer the first question, we need population-based, mixed-gender European samples covering a time-span across childhood and adolescence, and advanced statistical methods to account for individual heterogeneity.

The second question we aim to answer is whether conduct problems are a specific adjustment problem or one of many symptoms of an underlying psychopathology, that is, indicative of overall adjustment issues later in life. Moreover, by examining how membership in the trajectory groups predicts future (mal)adjustment, the distinction between different trajectories (adolescence-onset vs. adolescence-limited, and childhood-limited vs. child-onset/life-course-persistent) can be empirically validated. Overall, studies have found that the child-onset/life-course-persistent group experiences the highest health problems, with adolescence-onset individuals faring only slightly better in late adolescence (Kretschmer et al. 2014) and adulthood (Miller et al. 2010; Odgers et al. 2007; Roisman et al. 2004). Specifically, adolescence-onset males showed heightened mental health problems, substance abuse, and financial problems in adulthood (Moffitt et al. 2002).

The childhood-limited group seems to fare better than the other two groups, but report heightened internalizing symptoms (Miller et al. 2010; Moffitt et al. 2002; Odgers et al. 2008). That is, although childhood-limited children desist in conduct problems, they might increase in other types of maladjustment such as internalizing problems (cf. Moffitt et al. 2008). This claim was not supported by findings from a direct test of this idea (Barker et al. 2010), but in this study the trajectories were terminated at age 13 and outcomes later in life are still unknown. Thus, it remains rather unclear to what extent childhood-limited children are adjusted in early adulthood. If the claim is true that these childhood-limited children may be worse off in adulthood, though distinctly to life-course-persistent and adolescence-onset, early intervention and prevention is required to the same extent. In sum, the above mentioned studies show that the distinction between conduct problem trajectories is theoretically and substantively relevant as they differently predict future adjustment. They also indicate that childhood conduct problems may point to a generic psychopathology expressed by different types of difficulties later in life.

## The Present Study

This study has two main goals: (1) validation of child conduct problem trajectories in a European population-based sample consisting of both boys and girls and using an age-span from early childhood to late adolescence, thus examining whether conduct problems are specific to a developmental period and (2) identifying (mal)adjustment of the trajectory groups in emerging adulthood, thus examining whether conduct problems are a specific adjustment problem or part of a more generic psychopathology. We applied latent class growth analysis, which derives trajectory membership empirically instead of using arbitrary cut-offs. We avoided same-reporter bias by using parent-reports of child conduct problems at six subsequent waves across age 4–17 and self-reports of (mal)adjustment in emerging adulthood at age 17–20. We hypothesized to find a life-course-persistent, childhood-limited, and adolescence-onset trajectory for both boys and girls next to a large group of abstainers (cf., Barker and Maughan 2009; Odgers et al. 2008).

Given the persistence of problems for the life-course-persistent group, we hypothesized that these individuals would show the highest and most generic maladjustment in emerging adulthood. It is difficult to speculate on the future functioning of the adolescence-onset and childhood-limited groups since theoretical claims about predictors and prognosis are not consistent with the empirical findings reviewed above. That is, Moffitt’s theory (1993) and Moffitt et al. (1996) suggests the ability to recover from childhood-limited conduct problems after childhood, and normativity of conduct problems in adolescence and the ability to desist from these problems in adulthood, thus theoretically both groups should be healthily adjusted in adulthood. However, previous studies found that both groups are at heightened risk for adult maladjustment—and internalizing problems in particular for the childhood-limited group—as compared to stable lows (Moffitt et al. 2002; Odgers et al. 2007, 2008). Hence, in the current study, we explored (mal)adjustment of these groups in emerging adulthood, hypothesizing adjustment of these groups to range between the stable low and life-course-persistent group (cf. Kretschmer et al. 2014).

## Methods

### Procedure and Participants

This study is part of the ongoing longitudinal Flemish Study on Parenting, Personality, and Development that started in 1999 (FSPPD; Prinzie et al. 2003) for which data were collected at seven measurement waves (in 1999, 2000, 2001, 2004, 2007, 2009, and 2012). In 1999, a stratified sample of elementary-school-aged children attending regular schools in Belgium (Western Europe) was randomly selected. Strata were constructed according to geographical location (province), gender, and age. All participants had the Belgian nationality. Details on recruitment and procedure are described in Prinzie et al. (2003). All participants took part voluntarily and confidentiality was guaranteed. All participants gave written informed consent.

Our sample involved the children for whom mothers provided information. At the first assessment (T1), the total sample consisted of 682 mothers (92.5 % two-parent families). The number of children living at home ranged from one to seven (mean 2.4). Target children’s ages ranged between 4 and 7 years old at (*M* = 5 years 7 months, *SD* = 1.16) and 49.5 % were boys. The mean age of the mothers was 33 years 11 months (range 24 years 1 month–49 years; SD = 3.64). Most mothers (45 %) were educated to non-university higher education (comparable to community college).

At the subsequent assessments, the number of participants were as follows: 616 (age range 5–8 years, 48.9 % boys) at T2; 595 (age range 6–9 years, 50.3 % boys) at T3; 518 (age range 9–12 years, 49.3 % boys) at T4; 478 (age range 12–15 years, 47.4 % boys) at T5; and 437 (age range 14–17 years, 47.4 % boys) at T6. These data were used to examine the conduct problems trajectories. We used Full Information Maximum Likelihood estimation (see “Analytic Strategy” section) to include all 682 participants in the trajectory analyses.

At the final assessment (T7), 434 individuals (age range 17–20 years, 47 % boys) reported on their (mal)adjustment. Of these 434 individuals, 79 % still lived at home with their parents, whereas the remaining participants lived in lodgings and spent most of their weekends at home with their parents (20 %) or lived on their own and/or with a partner (1 %).

### Measures

#### Conduct Problems

Child conduct problems were rated by mothers at T1–T6 with the Dutch translation of the Child Behavior Checklist (CBCL, Achenbach 1991a; Verhulst et al. 1996). The DSM oriented subscale Conduct Problems (Achenbach et al. 2003) consists of 16 items at T1–T4 (CBCL/4–18) and 17 items at T5–T6 (CBCL/6–18) and comprises behaviors such as fighting, fire setting, truancy, and stealing. Each item was rated as 0 (*not true*), 1 (*somewhat/sometimes true*), or 2 (*very/often true*) evaluating the past 6 months. To create a Conduct Problems subscale, at each measurement wave the item scores were summed and for T1–T4 subsequently multiplied by 17/16 (cf. Achenbach and Rescorla 2001) due to a one item difference resulting from version changes between CBCL/4–18 and CBCL/6–18. Cronbach’s alphas for the Conduct Problems subscale ranged from .70 to .81. Given that our sample is population-based, only few participants ranked in the clinical range for conduct problems (ranging from 4 to 21 participants across waves). Therefore, a clinical cut-off (i.e., 98th percentile) was not feasible as the number of observations per category would be too low to analyze. Instead we used quartile scores to facilitate model convergence in the estimation of the trajectories.

#### Outcomes in Emerging Adulthood

At T7, maladjustment was measured with the Youth Self-Report (YSR; Achenbach 1991b) using the same rating as for the CBCL. We created syndrome scores for Aggressive Behavior, Rule-breaking Behavior, Anxious/Depressed, Withdrawn/Depressed, Somatic Complaints, Thought Problems, Attention Problems, and Social Problems. For each subscale, a mean score was calculated and *z*-standardized to ease interpretation of the results. Reliabilities were acceptable with all Cronbach’s alphas between .66 and .90 with the exception of Rule-breaking (α = .54) and Attention Problems (α = .59).

In addition, social adjustment was measured by the Close Friendship subscale of the revised Self-Perception Profile for Adolescents (SPPA; Harter 1988). The revised SPPA, using one instead of two opposite statements per item, has shown to have adequate reliability and validity (Wichstraum 1995). Five items measured whether individuals are able to form and maintain friendships that are characterized by trust and self-disclosure, e.g., “I do not have a close friend I can share my secrets with” (reversely coded). The items were rated on a 6 point scale (1 = *not at all true*, to 6 = *completely true*). A mean score was calculated and subsequently z-standardized. Reliability of the Close Friendship subscale was good (α = .79).

### Analytic Strategy

We applied mixture modeling in Mplus 7.0 (Muthén and Muthén 1998) with Full Information Maximum Likelihood (FIML) estimation to examine the number and shape of conduct problem trajectories using the six measurements of conduct problems across age 4–17 years and accounting for the unequal time points. Solutions with different numbers of classes were compared using entropy (a measure of class separation with a value of 1.00 representing perfect separation where solutions with an entropy above .8 are preferred), Bayesian Information Criterion (BIC), which is used to select among non-hierarchical models and penalizes over-fitting (i.e., assuming too many classes), Lo–Mendell–Rubin Likelihood Ratio test (LMR LRT) and Bootstrapped Likelihood Ratio test (BLRT), which are used to compare a model with k classes against a model with k − 1 classes. Significant *p* values indicate that the model with k classes represents a better fit to the data than a model with k − 1 classes. Finally, we paid close attention to class size and interpretability. Given that our sample consists of both boys and girls we subsequently examined whether the same trajectory model would fit equally well for boys and girls. This was done by estimating the model with intercepts, slopes, and class distributions allowed to vary across gender which we compared to a model in which boys and girls were constrained to be equal. The model fits of gender-variant and gender-invariant models were compared using the Satorra–Bentler difference test.

Once the best fitting solution was established, we examined a range of outcomes to provide empirical evidence for the validity of the conduct problem trajectories. The auxiliary variables feature in Mplus (Asparouhov and Muthén 2014) allows for straightforward addition of distal variables. Associations between the latent classes and the distal variables are estimated in one step but without distal variables interfering with latent class derivation. For the distal outcomes, Mplus computes an overall association using Wald’s test as well as pairwise class comparisons between the auxiliary variable means and probabilities (Asparouhov and Muthén 2014).

### Attrition Analyses

The latent class growth analysis (LCGA) is based on all available information, but the test of associations between latent classes and (mal)adjustment includes only individuals with information on outcomes (434 emerging adults, 64 % of the original sample). Attrition did not differ by gender (χ^2^(1) = 2.39, *p* = .14) or mother-reported conduct problems at T1 (*t* (680) = −1.29, *p* = .20), T2 (*t* (614) = −1.64, *p* = .10), or T3 (*t* (593) = −1.44, *p* = .15). Individuals with missing outcome information scored higher on mother-reported conduct problems at T4 (*t* (516) = −2.66, *p* < .05), T5 (*t* (476) = −3.26, *p* < .05), and T6 (*t* (435) = −3.98, *p* < .05) than those who completed the outcome assessment. These differences may result in a more conservative test of associations between our latent classes and distal variables as the ones who can be hypothesized to fare worst at T7 were more likely to be missing.

## Results

### Descriptive Statistics

Descriptive statistics for the total sample as well as for boys and girls separately are reported in Table 1. Boys scored higher than girls on conduct problems across all six waves. Boys also scored higher than girls on rule-breaking behavior and withdrawn/depressed symptoms at T7. In contrast, girls scored higher than boys on anxious/depressed symptoms and somatic complaints at T7. There were no significant gender differences in scores on friendship competencies, social problems, attention problems, thought problems, or aggressive behavior at T7.

**Table 1:** Descriptive statistics for conduct problems (T1–T6) and outcomes in emerging adulthood (T7)

	Total			Boys		Girls		*t*	df
	Mean	SD	N	Mean	n	Mean	n		
Conduct problems T1	1.64	2.30	682	2.21	339	1.08	343	6.57**	680
Conduct problems T2	1.36	2.00	616	1.79	300	0.93	316	5.41**	614
Conduct problems T3	1.31	2.19	595	1.72	298	0.90	297	4.63**	593
Conduct problems T4	1.08	1.96	518	1.47	255	0.70	263	4.56**	516
Conduct problems T5	1.22	2.04	478	1.46	228	1.01	250	2.44*	476
Conduct problems T6	1.26	2.34	437	1.64	207	0.91	230	3.29**	435
Externalizing problems									
Aggressive behavior	0.31	0.21	430	0.32	204	0.30	226	0.93	428
Rule-breaking behavior	0.43	0.19	430	0.48	204	0.40	226	4.26**	428
Internalizing problems									
Anxious/depressed	0.46	0.35	430	0.40	204	0.51	226	−3.49**	428
Withdrawn/depressed	0.33	0.31	430	0.37	204	0.30	226	2.31*	428
Somatic complaints	0.30	0.30	430	0.20	204	0.38	226	−6.54**	428
Thought problems	0.41	0.27	430	0.43	204	0.38	226	1.85	428
Attention problems	0.64	0.30	430	0.67	204	0.62	226	1.51	428
Social problems	0.34	0.25	430	0.33	204	0.34	226	−0.28	428
Friendship competency	5.10	0.84	434	5.05	207	5.16	227	−1.33	432

Descriptive statistics are based on raw scale scores (continuous and unstandardized)

* *p* < .05; ** *p* < .01

### Conduct Problem Trajectories

**Fig. 1:**
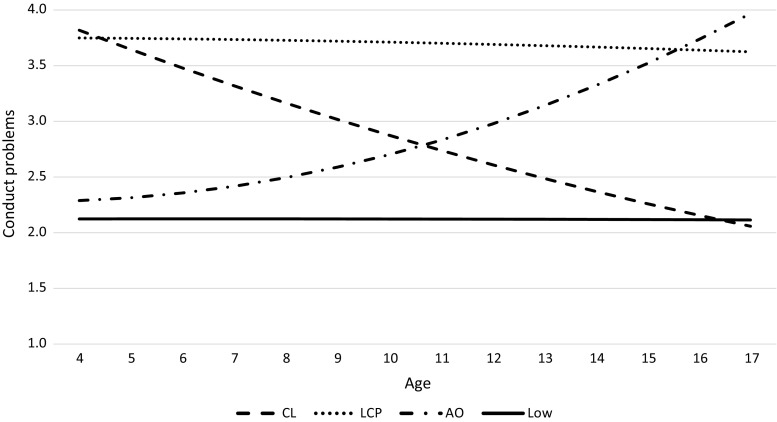
Conduct problem trajectories between age 4 and 17 years. *CL* childhood limited, *AO* adolescence onset, *LCP* life course persistent

Table 2 depicts fit statistics for models with increasing number of classes, showing that BIC decreased the more classes were added and entropy was satisfactory in all models. LMR-LRT indicated significant improvement in fit for the two- compared to the one-class model up until the four- compared to the three-class model. The five- class model did not add to the fit compared to a four-class model. The two-, three-, and four-class solutions yielded adequately large classes whereas the five-class solution yielded one very small class (1 % of sample). We thus concluded that the four-class solution fit the data best. There was no significant difference in model fit between gender-variant and gender-invariant models (TRd = 12.51; based on *∆df* = 12 and *χ* ^2^ critical value = 21.03), we thus retained the more parsimonious gender-invariant model. Figure 1 depicts the four conduct problems trajectories across age, labeled as Low (48 %; 40 % boys), Childhood Limited (CL; 12 %; 52 % boys), Adolescence Onset (AO; 15 %; 53 % boys), and Life Course Persistent (LCP; 25 %; 66 % boys).

**Table 2:** Model fit comparisons for increasing numbers of conduct problem trajectories

	BIC	Entropy	LMR-LRT	BLRT	Class sizes (%)
1 class	9495.14				
2 classes	8614.80	.84	872.99, *p* < .001	906.44, *p* < .001	34, 66
3 classes	8509.11	.78	126.93, *p* < .05	131.79, *p* < .05	11, 29, 60
4 classes	8304.13	.82	222.55, *p* < .05	231.08, *p* < .05	12, 15, 25, 48
5 classes	8326.27	.85	28.44, *p* = .42	29.53, *p* = .41	1, 12, 15, 25, 47
6 classes	7917.99	.84	91.94, *p* = .61	93.70, *p* = .60	7, 11, 13, 14, 15, 40

Class sizes are based on most likely class membership given posterior probabilities

*BIC* Bayesian information criterion, *LMR-LRT* Lo–Mendell Rubin likelihood ratio test, *BLRT* bootstrapped likelihood ratio test

### Prediction of Outcomes by Trajectories

Next, we examined whether the four conduct problem trajectories were differently associated with a range of variables indicative of (mal)adjustment in emerging adulthood. Important to note again, is that due to attrition at T7 the class sizes are smaller in these analyses than in the original trajectory analyses. Associations between latent classes and outcomes, including the number of participants per class, are presented in Table 3.

**Table 3:** Mean differences in adjustment variables in emerging adulthood by class membership

Outcome at T7	Class membership^1^				Overall Wald’s test	Significant class differences
	Low *n* = 198	CL *n* = 64	AO *n* = 78	LCP *n* = 94		
Externalizing problems						
Aggressive behavior	−0.21^a^	0.19^b^	0.10^b^	0.25^b^	χ (3) = 18.45, *p* < .01	CL, AO, LCP > L
Rule-breaking behavior	−0.21^a^	0.13^b^	0.22^b^	0.18^b^	χ (3) = 17.20, *p* < .01	CL, AO, LCP > L
Internalizing problems						
Anxious/depressed	−0.03^a^	0.20^a^	−0.04^a^	−0.04^a^	χ (3) = 2.58, *p* = .46	None
Withdrawn/depressed	−0.13^a^	0.16^b^	−0.02^ab^	0.18^b^	χ (3) = 7.49, *p* = .06	CL, LCP > L
Somatic complaints	−0.03^a^	0.05^a^	−0.06^a^	0.07^a^	χ (3) = 0.98, *p* = .81	None
Thought problems	−0.17^a^	0.29^b^	0.04^ab^	0.15^b^	χ (3) = 13.60, *p* < .01	CL, LCP > L
Attention problems	−0.14^a^	0.05^ab^	0.05^ab^	0.23^b^	χ (3) = 9.10, *p* < .05	LCP > L
Social problems	−0.14^a^	0.08^ab^	0.08^ab^	0.19^b^	χ (3) = 8.14, *p* < .05	LCP > L
Friendship competency	0.07^a^	−0.07^a^	−0.13^a^	0.04^a^	χ (3) = 2.50, *p* = .48	None

All outcome variables are *z*-standardized. Means with different (no overlapping) superscripts are significantly different from each other at *p* < .05

*L* low, *CL* childhood limited, *AO* adolescence onset, *LCP* life course persistent

^1^Most likely class membership given posterior probabilities

Overall, the Low class was most positively adjusted in emerging adulthood whereas the LCP class was least positively adjusted. In detail, we found that AO, CL, and LCP classes scored significantly higher on aggression and rule-breaking behavior than the Low class. Surprisingly, no significant differences emerged between LCP and AO or CL classes in these behaviors. In addition, the LCP and CL classes tended to score higher on withdrawn/depressed symptoms than the Low class, given that the overall Wald’s test was only marginally significant at *p* = .06. For thought problems, CL and LCP classes scored significantly higher than the Low class, whereas AO did not differ significantly from any other class. For attention problems the only significant difference was between the LCP and Low class, and no differences were found between LCP and AO or CL. Lastly, social adjustment was less favorable in the LCP class as compared to the Low class, whereas AO and CL did not differ significantly from Low or LCP. There were no significant differences across all four classes in anxious/depressed symptoms, somatic complaints, and friendship competencies.

## Discussion

The present study examined the onset and outcomes of child conduct problem trajectories in a European population-based sample of boys and girls covering early childhood until emerging adulthood to answer two important questions: Are conduct problems specific to a developmental period and are they specific as adjustment problem or indicative of generic maladjustment? Overall, our results corroborate the distinction between childhood-onset and adolescent-onset conduct problems (cf. Moffitt 1993) but also provide evidence for an extension of this taxonomy to distinguish between life-course-persistent and childhood-limited trajectories (cf. Barker and Maughan 2009; Odgers et al. 2008). Interestingly, although boys were overrepresented in the life-course-persistent, childhood-limited, and adolescence-onset trajectories, no gender differences emerged in the estimation of these trajectories. This means that the display of conduct problems develops in similar ways among boys and girls, consistent with findings in the Dunedin sample (Odgers et al. 2008) and in the ALSPAC sample of early adolescents (Barker and Maughan 2009).

In contrast to some previous studies, the age span of our study enabled us to clearly distinguish childhood-limited from life-course-persistent, and to a lesser extent also between adolescence-limited and adolescence-onset groups since the age 17 is not quite yet the “end” of adolescence. Members of the childhood-limited trajectory showed similarly high levels of conduct problems in early childhood as the life-course-persistent group, but dropped to a level similar to the Low group in adolescence. The turning point seems to lie around age 10, where the opposite patterns of the childhood-limited and the adolescence-onset trajectory intersect. That is, the adolescence-onset group started off at similarly low levels of conduct problems as the Low group and increased from about age 10, displaying comparable conduct problem levels as the life-course-persistent group by mid-adolescence. Thus, a childhood onset of conduct problems does not necessarily mean that these problems continue into adolescence and beyond—although the question remains whether or not desistance from these conduct problems (i.e., a childhood-limited pattern) goes hand in hand with emergence of other adjustment problems, which we will come back to later.

It is notable that our life-course-persistent group was relatively large (25 %) compared to other studies (approximately 10 %; e.g., Barker and Maughan 2009; Odgers et al. 2008). The reason for this difference might be our measure of conduct *problems* instead of conduct *disorder* (clinical diagnosis) in a normative population-based sample, and because we did not use a clinical cut-off (unlike Barker and Maughan 2009) as only very few individuals ranked in the clinical range. However, the shape and patterns of all our trajectories are similar to other—both clinical and cohort—studies. Interestingly, the adolescence-onset group consisted of only 15 % of our sample, which puts into question the normativity of adolescent conduct problems and consequently the maturity gap as a widely acknowledged explanation for this (Moffitt 1993). That is, our study showed that within a population-based sample it does not seem normative to display conduct problems in adolescence only (c.f., Piquero 2008), which makes it unlikely that these adolescents experience a maturity gap or, alternative, that the maturity gap is not directly associated with displaying conduct problems.

Related to this, our results suggest that the group we labeled as “adolescence onset” may actually start developing conduct problems well before adolescence. Our labeling is consistent with previous studies and theories regarding the onset of conduct problems but, although the lines of the childhood-limited and adolescence-onset intersect around age 10, it seems that the true “onset” of conduct problems in the latter group lies before this age. The normativity of having some conduct problems may be debated here, but nevertheless it is worth mentioning. Future research should aim to shed more light on this topic; for example, it has been suggested that the extent to which a maturity gap is experienced depends on how parents interact with their children (e.g., level of autonomy granting and involvement in decision-making), which is presumably culture-dependent (Kretschmer et al. 2015). There might be other explanations for the adolescence-onset of conduct problems, and more research should look into the appropriateness of this label. Moreover, the adolescence-onset group does not appear to desist from conduct problems in mid- or late adolescence, suggesting that the original adolescence-limited label may indeed be misleading. Should we worry about these adolescents when they enter adulthood? This question brings us to the second goal of our study.

Overall, we found that the life-course-persistent group was least favorably adjusted in emerging adulthood, showing the highest amount of externalizing-, attention-, thought-, and social problems. This is consistent with earlier studies into adjustment of the life-course-persistent group in adolescence (Kretschmer et al. 2014) and adulthood (Miller et al. 2010; Odgers et al. 2007; Roisman et al. 2004). Our findings could point to expression of the p factor, a general psychopathology factor that represents “the tendency to experience psychiatric problems as persistent and comorbid” (Caspi et al. 2014, p. 131). This general psychopathology factor represents not only duration and severity of problems, but also the cumulating risks early in life up until adjustment impairment in multiple aspects of adult life (Caspi et al. 2014), consistent with Moffitt’s (1993) theory on the life-course-persistent group. It should be noted, however, that in the externalizing domain (aggressive and rule-breaking behavior) the life-course-persistent group did not show significantly greater problems than adolescence-onset or childhood-limited groups and on some outcomes it only (negatively) differed from the low group. More research covering an even broader age range is necessary to conclude about the possibility of expression of the p factor and about the future functioning of this group, including serious forms of offending.

Interestingly, the adolescence-onset group showed similar levels of aggressive and rule-breaking behavior in emerging adulthood as the life-course-persistent group, which highlights the continuity of conduct problems in the adolescence-onset group beyond the adolescent period. Nevertheless, one could debate about the actual “ending” of adolescence; in the current study we labeled the ages 17–20 years as “emerging adulthood” in line with other studies, but it remains to be empirically tested whether the problems are limited or not to the adolescent period. All in all, in contrast to the life-course-persistent group, for the adolescence-onset group conduct problems seem to be a specific adjustment problem that do not represent an underlying psychopathology as there were no abnormalities in internalizing, thought, or social problems in emerging adulthood. Further follow up is needed to see what will happen later in life when these adolescents progress further into adulthood and future studies might do well to investigate this in more detail. In short, conduct problems with an onset in adolescence are probably not normative and warrant early intervention to avoid the risk of behavioral maladjustment in (emerging) adulthood.

The adjustment of the childhood-limited group in emerging adulthood was most explorative and, therefore, also most interesting given the mixed results in previous research (Moffitt et al. 2008). Although the conduct problems of the childhood-limited group are by definition limited to childhood, these children tended to be at heightened risk for withdrawn/depressed symptoms and thought problems in emerging adulthood. This finding is in line with results of the childhood-limited Dunedin males as reported in Moffitt et al. (2002), and our study shows that this also applies to females in a European sample. Future research might do well to look into this in more detail to find out what it is that makes these children more likely to suffer from internalizing problems later in life, especially as these problems are easily overlooked by their social environment. Could it be that an underlying “risk” is being expressed differently depending on the age? To answer this question, information for this specific group is needed both on a range of risk factors early in life as well as developmental outcomes later in life.

In addition, we would like to raise some generic issues inherent to estimating trajectory analyses that should be taken into account when reviewing the results of the current study (as well as future research). Identification of trajectories that are lower in prevalence will be more difficult in smaller samples (e.g., like our sample) as compared to larger sample sizes, because the lower prevalence trajectories might get collapsed in the more prevalent trajectories. In our study, the six-class solution fit the data worse than the four-class solution but this might be due to the low number of cases in some of these classes (i.e., 7 % of the sample). Thus, researchers using larger datasets might find evidence for more or differently shaped trajectory classes, although a review study on group-based trajectory modeling of externalizing problems did not find evidence of sample size being related to the number of classes found (Van Dulmen et al. 2009). Related to this issue, one should keep in mind that even within the four trajectories we observed there can exist some individual variation. That is, the trajectories as depicted in Fig. 1 show the average trend by grouping individuals that show comparable but not necessarily identical growth patterns, thus individuals within the groups can follow trajectories that deviate from the average trend (see e.g., Bushway et al. 2009). Future research should expand on this issue by examining in more detail the substantive and empirical validation of heterogeneity between as well as within trajectories, by looking at individual and social/contextual factors during the relevant period in time (i.e., adolescence in the adolescence-onset trajectory, or early childhood for the childhood-limited group) that may either reinforce or mitigate existing behavioral patterns.

Of course, the results of this study should be interpreted in the context of some limitations. First, not surprisingly given the time-span of 14 years, our study had to deal with attrition. Attrition analyses showed that attrition was related to amount of conduct problems, meaning that individuals with severe levels of conduct problems were more likely to be missing at the final assessment. Consequently, the reported associations between trajectory membership and adult outcomes are likely to have been mitigated. Second, in order to prevent same-reporter bias we have used different reporters for conduct problems across childhood and adolescence (parent-reports) and outcomes in emerging adulthood (self-reports) but this means that maladjustment in emerging adulthood may have been under-reported due to response bias. In addition, our outcome measures were limited to behavioral (mal)adjustment and future studies should try to include a wider spectrum of adult functioning including work, school, and family-related indicators. Lastly, although our sample stretched from early childhood up until emerging adulthood, the age range still does not exclude a possibility for some adolescence-onset youth to desist from conduct problems and related maladjustment later in life. Thus, more studies using a greater time-span are needed to extend the research on this specific trajectory group.

## Conclusion

Our study has tried to make a significant contribution to the extant literature by examining the shape and outcomes of conduct problem trajectories in a European population-based sample of boys and girls followed from early childhood into emerging adulthood and by using advanced statistical methods. We can conclude that adolescence-onset conduct problems may be more serious and less normative than previously believed and that no matter the trajectory they followed, all children who displayed conduct problems were worse off in emerging adulthood as compared to abstainers, although the extent to which their maladjustment was generic or specific to conduct problems differed among the trajectories. Interventions, therefore, should be aimed not only at the detection of conduct problems early in life but also at adolescents to avoid maladjustment later in life.

## References

[CR1] Achenbach TM (1991). Manual for the Child Behavior Checklist/4–18 and 1991 profiles.

[CR2] Achenbach TM (1991). Manual for the youth self-report and 1991 profiles.

[CR3] Achenbach TM, Dumenci L, Rescorla LA (2003). DSM-oriented and empirically based approaches to constructing scales from the same item pools. Journal of Clinical Child and Adolescent Psychology.

[CR4] Achenbach TM, Rescorla LA (2001). The manual for the ASEBA school-age forms & profiles.

[CR5] Asparouhov T, Muthén B (2014). Auxiliary variables in mixture modeling: Three-step approaches using Mplus. Structural Equation Modeling: A Multidisciplinary Journal.

[CR6] Barker ED, Maughan B (2009). Differentiating early-onset persistent versus childhood-limited conduct problem youth. American Journal of Psychiatry.

[CR7] Barker ED, Oliver BR, Maughan B (2010). Co-occurring problems of early onset persistent, childhood limited, and adolescent onset conduct problem youth. Journal of Child Psychology and Psychiatry.

[CR8] Broidy LM, Tremblay RE, Brame B, Fergusson D, Horwood JL, Laird R (2003). Developmental trajectories of childhood disruptive behaviors and adolescent delinquency: A six-site, cross-national study. Developmental Psychology.

[CR9] Bushway SD, Sweeten G, Nieuwbeerta P (2009). Measuring long term individual trajectories of offending using multiple methods. Journal of Quantitative Criminology.

[CR10] Caspi A, Houts RM, Belsky DW, Goldman-Mellor SJ, Harrington H, Israel S (2014). The p factor: One general psychopathology factor in the structure of psychiatric disorders?. Clinical Psychological Science.

[CR11] Fairchild G, Goozen SH, Calder AJ, Goodyer IM (2013). Research review: Evaluating and reformulating the developmental taxonomic theory of antisocial behaviour. Journal of Child Psychology and Psychiatry.

[CR12] Harter S (1988). Manual for the self-perception profile for adolescents.

[CR13] Kretschmer T, Dijkstra JK, Veenstra R, Blokland AAJ, Van der Geest VR (2015). Social and individual antecedents of adolescent-onset conduct problem behaviour. Routledge international handbook of criminal careers and life-course criminology.

[CR14] Kretschmer T, Hickman M, Doerner R, Emond A, Lewis G, Macleod J (2014). Outcomes of childhood conduct problem trajectories in early adulthood: Findings from the ALSPAC study. European Child and Adolescent Psychiatry.

[CR15] Miller S, Malone PS, Dodge KA, Conduct Problems Prevention Research Group (2010). Developmental trajectories of boys’ and girls’ delinquency: Sex differences and links to later adolescent outcomes. Journal of Abnormal Child Psychology.

[CR16] Moffitt TE (1993). Adolescence-limited and life-course-persistent antisocial behavior: A developmental taxonomy. Psychological Review.

[CR17] Moffitt TE, Arseneault L, Jaffee SR, Kim-Cohen J, Koenen KC, Odgers CL (2008). Research review: DSM-V conduct disorder: Research needs for an evidence base. Journal of Child Psychology and Psychiatry.

[CR18] Moffitt TE, Caspi A, Dickson N, Silva P, Stanton W (1996). Childhood-onset versus adolescent-onset antisocial conduct problems in males: Natural history from ages 3 to 18 years. Development and Psychopathology.

[CR19] Moffitt TE, Caspi A, Harrington H, Milne BJ (2002). Males on the life-course-persistent and adolescence-limited antisocial pathways: Follow-up at age 26 years. Development and Psychopathology.

[CR20] Muthén LK, Muthén BO (1998). Mplus user’s guide.

[CR22] Nagin DS, Farrington DP, Moffitt TE (1995). Life-course trajectories of different types of offenders. Criminology.

[CR23] Nagin DS, Land KC (1993). Age, criminal careers, and population heterogeneity: Specification and estimation of a nonparametric, mixed Poisson model. Criminology.

[CR24] Odgers CL, Caspi A, Broadbent JM, Dickson N, Hancox RJ, Harrington H (2007). Prediction of differential adult health burden by conduct problem subtypes in males. Archives of General Psychiatry.

[CR25] Odgers CL, Moffitt TE, Broadbent JM, Dickson N, Hancox RJ, Harrington H (2008). Female and male antisocial trajectories: From childhood origins to adult outcomes. Development and Psychopathology.

[CR26] Piquero AR, Liberman AM (2008). Taking stock of developmental trajectories of criminal activity over the life course. The long view of crime: A synthesis of longitudinal research.

[CR27] Piquero AR, Brame R, Moffitt TE (2005). Extending the study of continuity and change: Gender differences in the linkage between adolescent and adult offending. Journal of Quantitative Criminology.

[CR28] Prinzie P, Onghena P, Hellinckx W, Grietens H, Ghesquiere P, Colpin H (2003). The additive and interactive effects of parenting and children’s personality on externalizing behaviour. European Journal of Personality.

[CR29] Roisman GI, Aguilar B, Egeland B (2004). Antisocial behavior in the transition to adulthood: The independent and interactive roles of developmental history and emerging developmental tasks. Development and Psychopathology.

[CR30] Van Dulmen MH, Goncy EA, Vest A, Flannery DJ, Savage J (2009). Group-based trajectory modeling of externalizing behavior problems from childhood through adulthood: Exploring discrepancies in the empirical findings. The development of persistent criminality.

[CR31] Verhulst FC, van der Ende J, Koot HM (1996). Handleiding voor de CBCL/4–18.

[CR32] Wichstraum L (1995). Harter’s self-perception profile for adolescents: Reliability, validity, and evaluation of the question format. Journal of Personality Assessment.

